# Comparison of Anthropometric and Cephalometric Measurements Obtained by Stereophotogrammetry and 3D Computed Tomography of the Nose Before Septorhinoplasty

**DOI:** 10.1007/s00266-024-04097-9

**Published:** 2024-05-16

**Authors:** Ali Can Aydin, Percin Karakol, Agit Sulhan, Hamdullah Erk, Mehmet Bozkurt

**Affiliations:** 1 Department of Plastic, Reconstructive, and Aesthetic Surgery, Basaksehir Cam and Sakura City Hospital, Istanbul, Turkey; 2 Department of Radiology, Cemil Tascioglu City Hospital, Istanbul, Turkey; 3 Department of Plastic, Reconstructive and Aesthetic Surgery, Health Science University Bagcilar Education and Training Hospital, Istanbul, Turkey

**Keywords:** Rhinoplasty, Stereophotogrammetry, Computed tomography

## Abstract

**Introduction:**

Computed tomography (CT) is normally used in evaluation of patients with esthetic and functional nasal deformities. Stereophotogrammetry (SPG) is a measurement device that is an alternative to CT and does not harm human health. In this single-center retrospective study, we aimed to evaluate measurements obtained with CT and SPG.

**Methods:**

The measurements of 18 patients who applied to our clinic between January 2022 and August 2022 and planned for septorhinoplasty were performed on both 3D images obtained with paranasal sinus CT and SPG device (SLR type Vectra H1 system). Measurements included that dorsocolumellar length, columella-filtral length, nasal tip projection ratio (dorsocolumellar length/columella-filtral length), columella-labial angle, nasofrontal angle, tip deviation direction, tip deviation angle, tip deviation distance and dorsal nasal hump.

**Results:**

Most of patients were male (61.1%). Mean age was 24.5 years. Only columella-labial angle measurements showed a low level of significant difference (*p* < 0.05). However, there was no significance difference in other measurements (*p* > 0.05). A significant strong correlation was observed between all Vectra and CT measurements (*p* = 0.000).

**Conclusion:**

SPG device can be applied quickly in polyclinic without giving radiation to patient. Measurements can be taken automatically using a software. Its use in postoperative period does not carry any risk. Disadvantage of SPG is lack of information about internal nasal passage. However, there is a strong correlation between measurements obtained from both measurement devices. Therefore, SPG can be considered as an alternative to CT imaging in operation planning.

**Level of Evidence IV:**

This journal requires that authors assign a level of evidence to each article. For a full description of these Evidence-Based Medicine ratings, please refer to the Table of Contents or the online Instructions to Authors www.springer.com/00266.

## Introduction

Functional and esthetic rhinoplasty is one of the most frequently applied plastic esthetic surgeries both in our country and in the world. The main goal is to repair the anatomical and functional disorders of the nose by minimally traumatizing the mucosal, cartilage and bone structures [1, 2]. For this purpose, detailed analysis should be done in the preoperative evaluation. Cartilage and bone structures should be defined in detail, and the operation should be performed in the light of this information [3]. Detailed preoperative evaluation should be performed to maintain the continuity of the dorsal esthetic lines in the deviated nose, to bring the nasal dorsum to the midline and to establish airway continuity. According to this evaluation, an operation management should be done carefully [4].

Even if the esthetic perception varies during the process, the long-term maintenance of the rhinoplasty result is the unchanging goal. After the operation, the situation that creates dissatisfaction for the surgeon and the patient is an undesirable change in postoperative outcomes. This situation has led physicians to develop the preoperative evaluation techniques and to find up-to-date methods [5]. For a high success in the rhinoplasty, it is crucial to make the detailed preoperative anthropometric and cephalometric measurements of the nose and to perform the operation plans accordingly [6].

Based on the standard imaging of the nose (frontal, lateral, oblique and basal), analyses for preoperative planning and postoperative evaluation are routinely performed in the clinics before and after rhinoplasty [7]. However, there are some inadequacies arising from the evaluation of a three-dimensional (3D) structure such as a nose on a two-dimensional (2D) image [8].

Various 3D techniques have been produced to demonstrate facial topography and to overcome the shortcomings of traditional photographic and radiographic methods. These imaging methods include 3D cephalometry, morphoanalysis, Moire topography, computed tomography (CT) assisted 3D imaging, 3D ultrasonography, 3D laser scanning and stereophotogrammetry (SPG) [9]. CT-assisted 3D imaging method is frequently used in our department, but this method exposes the patients to radiation as well as not a cost-effective technique [10]. Several research studies have assessed different computer imaging techniques and their capacity to effectively predict outcomes using computer simulations [11–13]. To date, a comparison of the anthropometric and cephalometric measurements obtained by SPG and 3D CT of the nose before septorhinoplasty has not been studied yet. Therefore, in this study, we aimed to evaluate the use of SPG measuring devices that do not harm human health in clinical applications, as an alternative to 3D CT obtained by multiplanar reconstruction method in preoperative evaluations of the nose in septorhinoplasty patients.

## Patients and Methods

### Patient Selection

In this retrospective single-center study, 18 patients who applied to our department with the complaints of serious functional and cosmetic nasal deformity and were planned for septorhinoplasty operation between January 2022 and August 2022 were included. Patient age ranges were between 18 and 39 years. Patients who had a previous nasal or facial surgery, dermal filler or botulinum toxin application and rope sling procedures were not included in the study. In addition, the sensitive population such as the disabled, children, pregnant women, postpartum women and lactating women, patients in the intensive care and unconscious were not included in the study. The study protocol was approved by the Ethical Committee of Non-invasive Clinical Research of Sisli Hamidiye Etfal Training and Research Hospital Health Application and Research Center (SUAM) of Health Sciences University (Decision No: 3744-2022/12/13). The study protocol was in accordance with the Helsinki Declaration.

### Clinical and Radiological Evaluation

In the preoperative period, photographs of 18 patients were taken with a Canon EOS 70° brand 50 mm lens camera under standard ambient lighting conditions in the photographic room in the clinic. SPG images were obtained with SLR type Vectra H1 system (Canfield Imaging, Parsippany, NJ, USA) and paranasal CT images were obtained from Siemens SOMATOM Definition Edge CT device with standard techniques. Images for each patient were converted to 3D images using the multiplanar reconstruction technique. Before the images were taken, the participants were asked to remove their jewelry and pull back the hair covering their forehead and ears so that all their faces could be seen clearly.

### Stereophotogrammetry

**Fig. 1:**
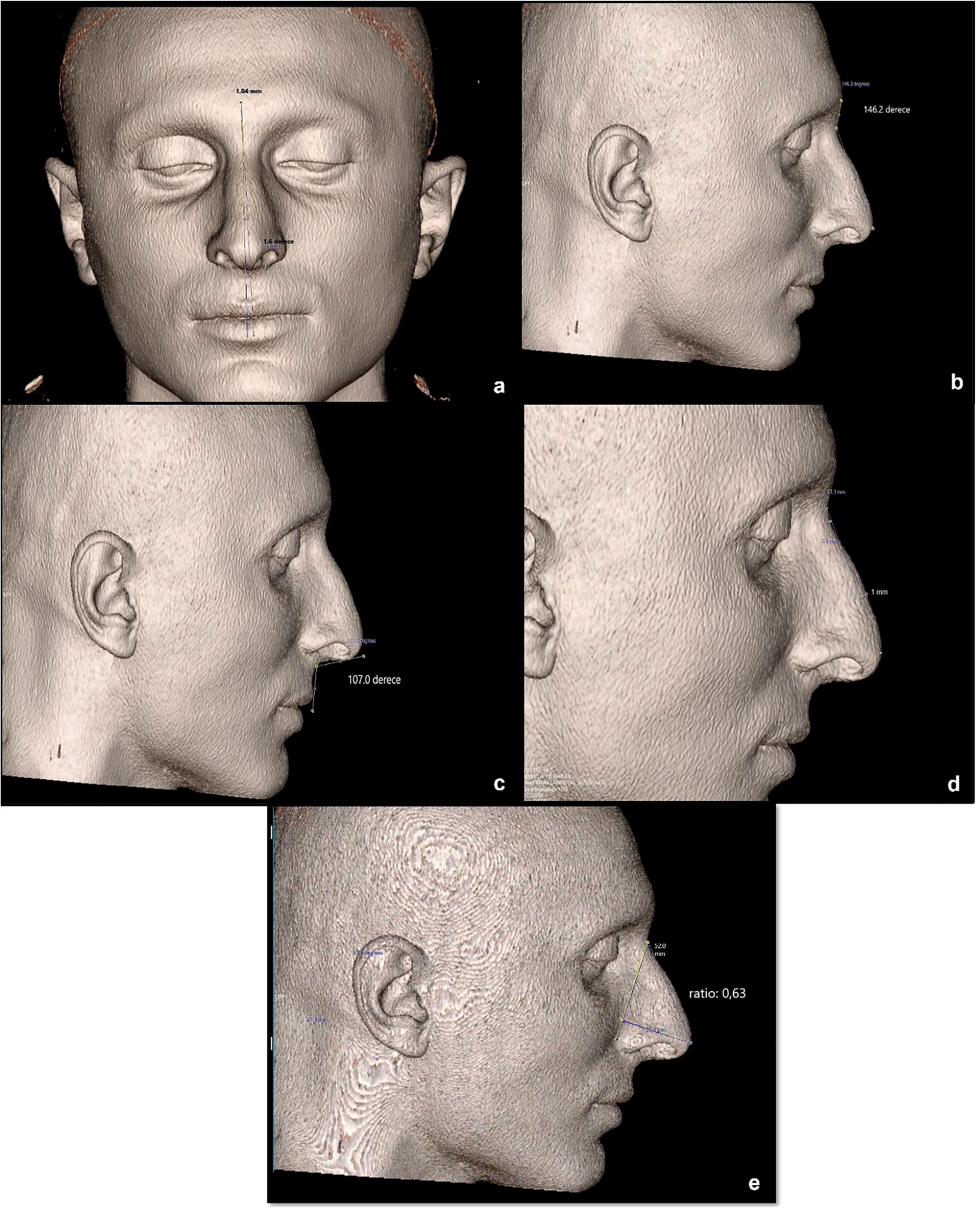
A patient photographed with stereophotogrammetry: (**a**) Tip deviation angle and distance measurement, (**b**) nasofrontal angle measurement, (**c**) columella-filtral angle measurement, (**d**) dorsal nasal hump measurement, (**e**) nasal tip projection ratio measurement

Multiple simultaneous photos were taken from different angles using SPG which consists of a single handheld Canon SLR camera body equipped with a dedicated lens and a range finder that allows 3D imaging. All patients were asked to maintain a neutral facial expression, and the imaging was initiated. For the first capture, the camera lens was lifted slightly upward, positioning the camera 45° to the right of the participant and approximately 30 cm below the patient’s face. The Vectra H1 system guided the user with visual prompts to ensure the camera was at the correct distance from the face target, with two mirrored green dots acting as guides. The camera distance was correct when the dots converged on the face surface. The first point target was the patient’s cheek (the intersection of the imaginary line descending from the lateral canthus and the horizontal imaginary lines drawn from the upper lip). The second photo capture was right in front of the patient; the camera was brought to an equal distance with the patient’s face and the green dot was taken at the midline of the face, aiming between the upper lip and the nose. The third capture was similar to the first capture; the camera lens was lifted slightly upward by positioning the camera 45° to the left side of patient and approximately 30 cm below the face. All images were recorded, and then three consecutive captures were transferred to a proprietary Vectra software to assemble a full 3D face model. By automatically identifying the overlapping face surface features between the right, middle and left 3D captures, the merging was performed automatically. Then, the data of the patients were automatically measured in the software and their numerical values were noted. These data included the dorsocolumellar length, columella-filtral length, nasal tip projection ratio (dorsocolumellar length/columella-filtral length), columella-labial angle, nasofrontal angle, tip deviation direction, tip deviation angle, tip deviation distance and dorsal nasal hump measurements (Fig. 1).

### CT Imaging

**Fig. 2:**
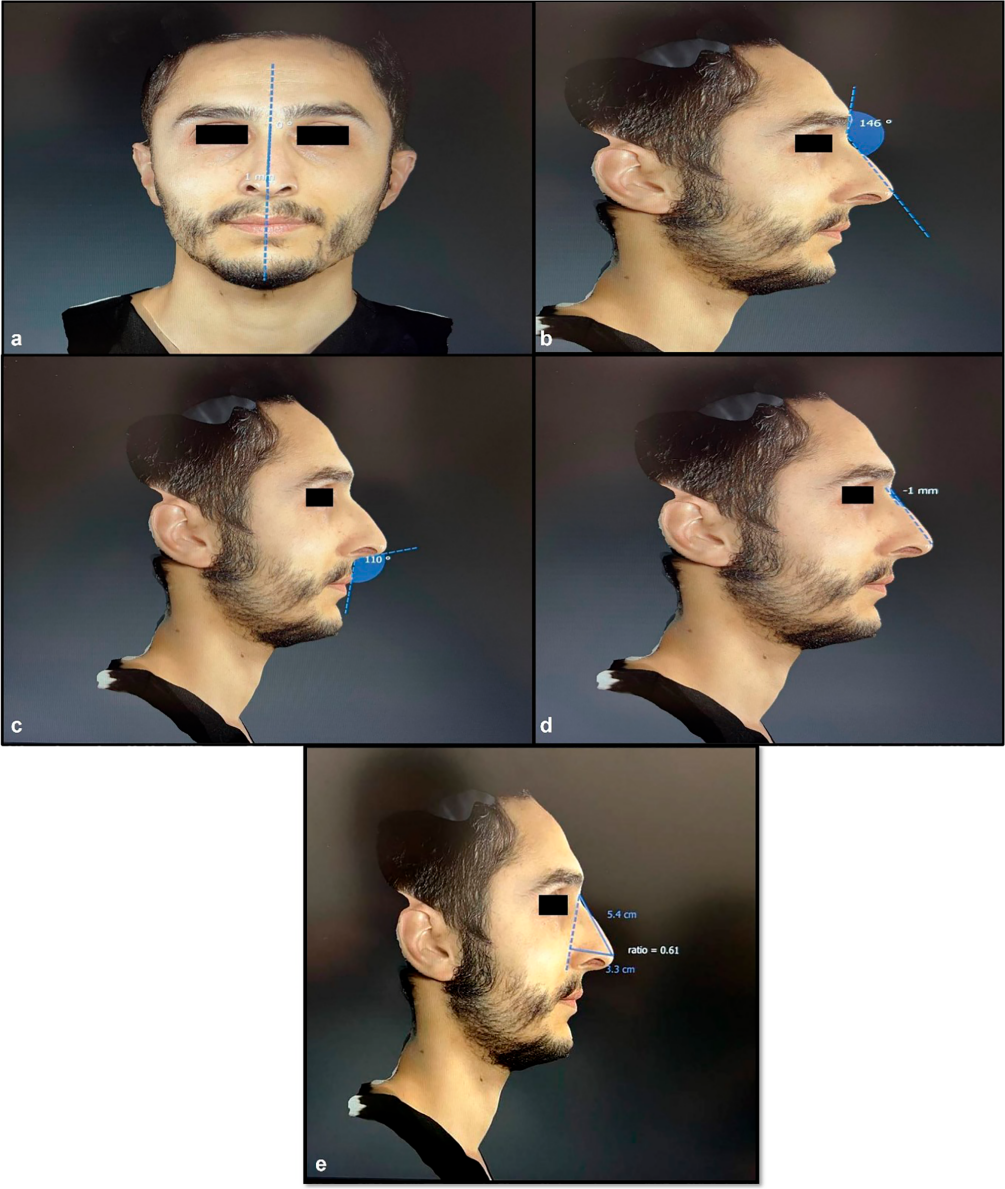
A patient photographed with three-dimensional computed tomography: (**a**) Tip deviation angle and distance measurement, (**b**) nasofrontal angle measurement, (**c**) columella-filtral angle measurement, (**d**) dorsal nasal hump measurement, (**e**) nasal tip projection ratio measurement

Preoperative evaluations were made on the paranasal sinus CT examinations obtained before the rhinoplasty operation of each patient. All CT images were obtained from Siemens SOMATOM Definition Edge CT device with standard techniques. Images for each patient were converted to 3D images using the multiplanar reconstruction technique and evaluated on the software (ExtremePACS, Turkey). On these images, the dorsocolumellar length, columella-filtral length, nasal tip projection ratio (dorsocolumellar length/columella-filtral length), columella-labial angle, nasofrontal angle, tip deviation direction, tip deviation angle, tip deviation distance and dorsal nasal hump were measured and all radiological measurements (Fig. 2) were compared with the measurements obtained by the SPG device.

### Follow-Up

All patients received similar postoperative care and followed up at least for 6 months. All patients were discharged on the 1st postoperative day. The nasal tampon and external splint were removed on the seventh day after surgery. No major complications developed in any of the patients during the follow-up period. Varying degrees of ecchymosis and edema occurred in all treatment groups. All of these resolved with compression and medical therapy.

### Statistical Analysis

SPSS 28.0 program was sued in all statistical analyses of the data. The descriptive statistics of the data included the mean, standard deviation, median minimum, maximum, frequency and ratio values. The distribution of variables was measured with the Kolmogorov–Smirnov test, and paired sample t-test and Wilcoxon test were used in the analysis of dependent quantitative data. An intraclass correlation analysis was used in the correlation analysis. A *p* value < 0.05 was considered statistically significant level.

## Results

Of 18 patients included in the study, 11 (61.1%) were male and 7 (38.9%) were female. The mean age of patients was 24.5 ± 6.3 years (min. 18–max. 39). The mean dorsocolumellar length was found to be 4.9  ± 0.4 cm in SPG and 4.8 ± 0.4 cm on CT measurements. The mean columella-filtral length was found to be 2.9 ± 0.3 cm in SPG and 3.0 ± 0.2 cm on CT measurements. The nasal tip projection ratio was 0.6° ± 0.1 in SPG and 0.6° ± 0.0 in CT measurements. The mean columella-labial angle was 109.1° ± 9.1 in SPG and 106.8° ± 10.1 in CT measurements. The mean nasofrontal angle was found to be 151.7° ± 6.7 in SPG and 150.7° ± 6.4 on CT measurements. The mean tip deviation angle and distance were 1.1° ± 1.0 and 0.8 ± 0.6 mm in SPG and 1.1° ± 0.6 and 0.9 ± 0.5 mm in CT measurements, respectively. The mean dorsal nasal hump was 2.2 ± 1.4 mm in SPG and 2.5 ± 1.2 mm on CT measurements (Table 1).

**Table 1:** Anthropometric and cephalometric measurements of the nose obtained by stereophotogrammetry (SPG) and computed tomography (CT)

No	Dorsocolumellar length (cm)		Columella-filtral length (cm)		Nasal tip projection ratio (°)		Columella-labial angle (°)		Nasofrontal angle (°)		Tip deviation angle (°)		Tip deviation distance (mm)		Dorsal nasal hump (mm)	
	SPG	CT	SPG	CT	SPG	CT	SPG	CT	SPG	CT	SPG	CT	SPG	CT	SPG	CT
1	4.50	4.60	2.80	2.90	0.62	0.63	110.0	110.0	152	156	2.00	2.00	1.00	1.00	2.00	3.00
2	5.30	4.90	2.60	2.70	0.49	0.55	115.	115.0	150	149	1.00	1.00	1.00	1.00	3.00	3.00
3	4.60	4.00	2.70	2.70	0.58	0.67	119.0	105.0	148	146	4.00	2.00	2.00	2.00	3.00	3.00
4	5.10	5.00	2.00	3.10	0.39	0.62	113.0	112.0	151	149	2.00	2.00	2.00	2.00	3.00	3.00
5	5.00	4.80	2.80	2.90	0.56	0.60	101.	99.	138	140	1.00	1.00	1.00	1.00	1.00	2.50
6	5.10	5.30	3.20	3.10	0.63	0.58	111.0	108.0	159	158	0.00	0.00	0.00	0.00	3.00	3.00
7	4.30	4.30	3.10	2.90	0.72	0.67	128.0	128.0	153	153	1.00	1.00	0.00	1.00	3.00	3.00
8	5.30	4.90	2.90	3.00	0.55	0.61	116.0	117.0	153	153	1.00	1.00	1.00	1.00	2.00	2.20
9	4.30	4.50	2.60	2.70	0.60	0.60	99.0	95.0	149	147	1.00	1.00	0.00	1.00	4.00	4.00
10	4.90	4.80	2.60	2.70	0.54	0.56	95.0	92.0	151	147	1.00	1.00	1.00	1.00	1.00	1.20
11	5.50	5.40	3.10	3.10	0.58	0.57	103.0	96.0	157	156	1.00	1.00	1.00	1.00	3.00	3.50
12	5.30	5.30	3.20	3.40	0.60	0.64	99.0	95.0	146	145	1.00	1.00	1.00	1.00	2.00	2.50
13	4.10	4.20	2.80	2.80	0.70	0.67	106.0	105.0	142	139	0.00	0.00	0.00	0.00	2.00	2.50
14	4.80	4.90	3.00	3.10	0.63	0.63	121.0	122.	156	156	1.00	1.00	1.00	1.00	2.00	1.80
15	4.50	4.40	2.90	3.10	0.66	0.70	99.00	99.0	152	152	2.00	2.00	1.00	1.00	1.00	1.10
16	5.10	5.10	3.20	3.10	0.62	0.60	102.0	102.0	164	161	1.00	1.00	0.00	1.00	3.00	2.00
17	5.10	5.20	2.90	3.00	0.57	0.57	116.0	116.0	163	159	0.00	0.00	0.00	0.00	4.00	4.00
18	5.40	5.20	3.30	3.30	0.61	0.63	110.0	107.0	146	146	0.00	1.00	1.00	1.00	1.00	1.00

*SPG* stereophotogrammetry, *CT* computed tomography

A significant difference was observed between the mean columella-labial angle measurements by SPG and CT (*p* = 0.019). However, no significant difference was observed in the dorsocolumellar length, columella-filtral length, the nasal tip projection ratio, nasofrontal angle, tip deviation angle and distance and dorsal nasal hump measurements by SPG and CT (*p* > 0.05) (Table 2).

**Table 2:** Comparison and correlation findings of stereophotogrammetry (SPG) and computed tomography (CT)

	SPG X ± SS, Median	CT X ± SS, Median	P value	Correlation r (95% Cl)	ICC P value
Dorsocolumellar length (cm)	4.9 ± 0.4 5.1	4.8 ± 0.4 4.9	0.149^E^	0.926 0.803–0.972	0.000
Columella-filtral length (cm)	2.9 ± 0.3 2.9	3.0 ± 0.2 3.0	0.114^E^	0.653 0.073–0.870	0.018
Nasal tip projection ratio (°)	0.6 ± 0.1 0.6	0.6 ± 0.0 0.6	0.153^E^	0.620 0.016–0.858	0.027
Columella-labial angle (°)	109.1 ± 9.1 110.0	106.8 ± 10.1 106.0	0.019^E^	0.963 0.902–0.986	0.000
Nasofrontal angle (°)	151.7 ± 6.7 151.5	150.7 ± 6.4 150.5	0.051^E^	0.976 0.935–0.991	0.000
Tip deviation angle (°)	1.1 ± 1.0 1.0	1.1 ± 0.6 1.0	0.655^W^	0.878 0.674–0.954	0.000
Tip deviation distance (mm)	0.8 ± 0.6 1.0	0.9 ± 0.5 1.0	0.083^W^	0.884 0.691–0.957	0.000
Dorsal nasal hump (mm)	2.2 ± 1.4 2.5	2.5 ± 1.2 2.8	0.101^W^	0.868 0.647–0.951	0.000

*SPG* stereophotogrammetry, *CT* computed tomography, ^S^ Paired sampling t-test, ^w^ Wilcoxon test, *ICC* in-class correlation

In the correlation analysis, all SPG and CT measurements were found to have a significantly strong correlation (*r* = 0.926/*p* = 0.000 for dorsocolumellar length; *r* = 0.653/*p* = 0.018 for columella-filtral length; *r* = 0.620/*p* = 0.027 for nasal tip projection ratio; *r* = 0.963/*p* = 0.000 for columella-labial angle; *r* = 0.976/*p* = 0.000 for nasofrontal angle; *r* = 0.878/*p* = 0.000 for tip deviation angle; *r* = 0.884/*p* = 0.000 for tip deviation distance; *r* = 0.868/*p* = 0.000 for dorsal nasal hump) (Table 2).

## Discussion

In this single-center retrospective study, we aimed to compare the measurements obtained with CT obtained by multiplanar reconstruction method and SPG used in the preoperative evaluation of patients with septorhinoplasty operation. We found that the dorsocolumellar length, columella-labial angle, nasofrontal angle and tip deviation angle measurements were higher with SPG while the columella-filtral length and dorsal nasal hump measurements were lower with SPG compared to CT. However, there was no significant difference in these measurements. Nasal tip projection ratio (0.6) and tip deviation distance (0.8 mm) were found to be similar in both measurement methods. Only, the columella-labial angle measurements showed a low level of significant difference. The correlation analyzes showed a statistically significant strong correlation in all SPG and CT measurements.

To achieve optimal outcomes in the rhinoplasty, the nasal deformities should be determined by preoperative analysis. These analyzes also need to be evaluated for correct surgical management. Then, the ideal nose to be created is decided by surgeon. Then, the necessary intraoperative steps are applied for the ideal measurements [14, 15]. There are many methods/devices used to evaluate the nose, each brings its own disadvantages. Morphoanalysis, for example, requires an expensive equipment and is time consuming, impractical for regular use [16]. Moire topography is also a time-consuming technique, requiring detailed lighting control [10, 17]. 3D Laser scanning does not visualize the soft tissue well, and the motion artifacts are high due to long image acquisition time of up to 20 s. In 3D Ultrasonography, movement during the data acquisition and pressure of the probe on the soft tissues cause image distortion [18]. In our study, we obtained anthropometric and cephalometric measurements of the nose with preoperative SPG and 3D CT methods. We confirmed that SPG can be applied more quickly and its use in the postoperative period does not pose any risk on the patient. We concluded that it should be considered as an alternative to CT imaging.

Preoperative CT can be used to ensure proper planning in the rhinoplasty and to accurately determine the defects of the nose before the operation [19]. Vital parameters evaluated in preoperative planning with 3D CT scanning are the factors included the nasal valve, alar and lateral cartilages, interdomal distance and bone structure which may improve the outcomes of septorhinoplasty [20, 21]. Sari et al. reported that preoperative CT parameters were found to be informative, but do not predict the postoperative success of septoplasty [21]. It is controversial whether preoperative CT is really necessary due to the radiation exposure and cost before the surgeries [22–24]. In addition, the metal objects in the oral cavity, if any, cause artifacts [25]. For this purpose, we investigated whether SPG could be used as an alternative to CT. And we found a strong correlation between CT and SPG in our results.

By the development of technology, the use of SPG method in the evaluation before the number of rhinoplasty operations has been increasing. The advantage is that it captures near snapshots (1.5 ms), which minimizes motion artifact. In addition, providing archived images for repeated analyses, collecting data in 3D format for further studies and obtaining high-resolution color images are other important advantages of SPG [26]. As validated in previous studies [27–29], it is a method with good accuracy and reproducibility to measure distances and volumes on the face, in addition to being able to obtain texture and color of the captured object. In this study, SPG measurements could be performed retrospectively and the measurements were compared with the data obtained with CT imaging. There was a low level of significant difference only in the columella-labial angle measurements, and there was no difference in other measurements between two methods. However, a strong correlation was observed between both methods in the correlation analysis, suggesting that SPG gave reliable outcomes as CT.

There are no reports on the comparison of the preoperative measurements by SPG and CT imaging modalities in rhinoplasty, specifically in the context of evaluating certain esthetic parameters of the nose, including dorsocolumellar length, columella-filtral length and nasal tip projection ratio. However, these parameters were previously evaluated in Turkish cohorts before [30-34]. In the study of Uzun et al., the mean dorsocolumellar length was found to be 55.26 mm and the mean columella-labial angle was 90.32° in a Turk cohort [30]. According to the report by Borman et al., the mean columella-labial angle was found to be 97.79° in Turkish men and 95.07° in Turkish women [31]. In our study, this length was calculated as 4.9 ± 0.4 cm in SPG measurement and 4.8 ± 0.4 cm in CT measurement. There was no significant difference between the dorsocolumellar length measurements by SPG and CT. However, it was noted that there was a strong correlation between these measurements. The columella-labial angle was found to be higher than the literature, which was measured as 109.1 ± 9.1° in SPG measurement and 106.8 ± 10.1° in CT measurements. A low statistically significant difference (*p* = 0.019) and a strong correlation (*r* = 0.963 *p* = 0.000) were observed between these measurements. In the study conducted by Karaca et al. in Turkey, the mean columella-filtral length was measured as 20.44 mm in men and 19.80 mm in women [32]. In our study, longer length was obtained compared to the study of Karaca et al. Columella-filtral length was calculated as 29 ± 3 mm in SPG measurement and 30 ± 2 mm in CT measurement, and a strong correlation was found with correlation analysis. However, no significant difference was observed between the measurements, suggesting that the SPG resulted in comparable outcomes for the columella-filtral length.

There were some limitations in our study. Eighteen patients included in our study were only a subset of the total rhinoplasties, and may have been prone to selection bias, impacted by compliance and follow-up. Despite the small sample size, a persuasive correlation was found between the two methods by correlation analysis. However, we could not have the data of the internal nasal passage with SPG. Despite manual soft-tissue landmark placement in addition to automated landmark placement with the Vectra software, the assessor-dependent errors could not be completely excluded. Another limitation was the absence of postoperative imagings in evaluating the outcomes of rhinoplasty procedures; however, our study was specifically designed to investigate an appropriate imaging method for preoperative decision-making, not to be an intraoperative guidance to achieve appropriate functional and cosmetic results including the morbidity of the procedure. Our focus was indeed on comparing preoperative measurements obtained from two different imaging modalities rather than assessing postoperative changes. While we understand the importance of before and after imaging in evaluating the outcomes of rhinoplasty procedures, the reason we compared the tomography and endoscopic methods in the study is to emphasize that tomography can be used as an imaging method in preoperative evaluation despite the fact that it contains X-rays, and it is even superior to endoscopy and rhinoscopy. However, we do not prefer tomographic imaging in the postoperative period because we do not find it logical since it already contains X-rays, and therefore, we think that the SPG method may be superior in the preoperative period.

## Conclusions

The present study indicated that the measurements made using SPG and CT techniques were comparable in preoperative evaluation of nose before septorhinoplasty, although there was a low difference in the mean columella-labial angle in between to methods. Moreover, a persuasive correlation was observed in the measurements by two techniques. Therefore, we believe that it can be considered as an alternative to CT imaging in the operation plan, since it can be applied quickly even in external areas outside the examination area, it is advantageous in patients with claustrophobia, its use in the postoperative period does not carry any risk and the measurements are similar to CT. As a future goal, we are planning to recruit a larger sample size including various groups of patients, evaluating the method’s reliability in subjects from a wider age range and in different races. Moreover, we will use these facial landmarks and anthropometric parameters to compare the differences in nasal morphology between ethnic groups, via including potential collaborations with other institutions.

## Data Availability

All data generated or analyzed during this study are included in this published article.

## Abbreviations

SPG: Stereophotogrammetry
CT: Computed tomography
3D: 3-Dimensional
2D: 2-Dimensional
